# Relationship between religiosity and smoking among undergraduate health sciences students

**DOI:** 10.47626/2237-6089-2019-0031

**Published:** 2021-02-26

**Authors:** Edson Zangiacomi Martinez, Carolina Cunha Bueno-Silva, Isabela Mirandola Bartolomeu, Livia Borges Ribeiro-Pizzo, Miriane Lucindo Zucoloto

**Affiliations:** 1 Faculdade de Medicina de Ribeirão PretoUniversidade de São PauloRibeirão PretoSPBrazil Faculdade de Medicina de Ribeirão Preto, Universidade de São Paulo (FMRP-USP), Ribeirão Preto, SP, Brazil.

**Keywords:** Religion and medicine, students, health occupations, smoking, healthy lifestyle

## Abstract

**Introduction:**

The university period is often characterized as a critical period of vulnerability for smoking habit initiation.

**Objective:**

The purpose of this cross-sectional study was to assess the relationship between religiosity and smoking among undergraduate students on health sciences courses.

**Methods:**

A total of 336 students on four health sciences courses (occupational therapy, speech therapy, nutrition, and physiotherapy) completed a cigarette smoking questionnaire along with the Duke University Religion Index.

**Results:**

Smoking prevalence was 8.3% among females and 12.7% among males. Prevalence among students who do not have a religion, but do believe in God, was higher than among those who do have a religion (16.3 and 6.3%, respectively). Organizational religious activity has a significant effect on smoking status.

**Conclusion:**

The students have health habits that are not only motivated by the technical knowledge acquired on their undergraduate courses, since there was a possible influence of social norms stimulated by religious institutions on their attitudes, knowledge and practices in health.

## Introduction

Age-standardized prevalence of daily smoking in 2015 among the overall Brazilian population was estimated at 8.2% among females and 12.6% among males.^1^ These percentages are relatively low when compared with those reported in countries such as Portugal (12.7 and 24.9%, respectively), Spain (18.6 and 25.6%), Switzerland (16.5 and 21.9%), and Argentina (14.6 and 21.1%), but similar to those found in the United States (11.7 and 14.4%).^1^ Although Brazil has experienced a large decline in smoking prevalence due to tobacco control interventions implemented in the last few decades,^2^ the direct annual cost of smoking to the public healthcare system is nonetheless estimated at 6.3 billion US dollars (at the prevailing exchange rate in 2019).^3^

The university period is often characterized as a critical period of vulnerability for smoking habit initiation.^4-6^ Smoking habits of students on health science courses deserve special consideration, since they presumably have reasonable knowledge about the harmful effects of tobacco and will become behavioral models, conveying the basic concepts of health into the community.^5,7^ In Brazilian studies that have been carried out in populations of undergraduate health sciences students, the smoking habit is found to be more prevalent among male students and is associated with variables such as the course semester attended, alcohol consumption,^6-9^ and use of antidepressants or anxiolytics.^10^

One factor that can protect university students from risky behaviors such as smoking is religiosity, as shown in a recent Iranian study.^11^ A number of other studies have also suggested that religiosity is associated with smoking habits in students from different cultural and ethnic backgrounds. For example, Nabipour et al.^12^ observed that waterpipe smoking has grown in popularity among youth and adolescents in Iran, but that religious observance may play a protective role in lowering waterpipe usage among Iranian university students. Isralowitz et al.^13^ found evidence that female Israeli college students who were more religious reported lower use of tobacco, alcohol, and other drugs. Pule et al.^14^ showed that intrinsic religiosity had effects on health risk behaviors, including smoking, among black university students in Limpopo, South Africa.

Considering university students, not much is known about religiosity’s protective role against smoking in the Brazilian context. In 2009, a population-based study entitled “First nationwide survey on the use of alcohol, tobacco and other drugs among college students in the 27 Brazilian state capitals” showed that use of tobacco was higher among students who did not attend religious groups or attended occasionally than among those attending religious services at least once a month.^15^ However, this study and others^5,7,15,16^ only considered religious affiliation and religious involvement as outcomes related to religiosity, but did not include other important dimensions, such as intrinsic religiosity. Therefore, the present cross-sectional study aims to investigate the prevalence of tobacco smoking and dimensions of religiosity measured by the Duke University religion index (DUREL) among undergraduate health sciences students at a Brazilian public university.

## Methods

### Participants and procedure

The study was conducted at the Ribeirão Preto campus of the Universidade de São Paulo, in Southeast Brazil, from August to November of 2018. Students on four health sciences courses (occupational therapy, speech therapy, nutrition, and physiotherapy) were interviewed in their classrooms during school hours with their teachers’ permission. Exclusion criteria were not adopted, in order to represent the full diversity of the characteristics of the student population. A printed questionnaire was used to collect data. All students gave their informed consent, and the study protocol was approved by the ethics committee at the Faculdade de Medicina de Ribeirão Preto.

### Instruments

The DUREL was used to measure religiosity.^17^ This five-item instrument assesses the following dimensions of religiosity: organizational religious activity (ORA), non-organizational religious activity (NORA), and intrinsic religiosity (IR).^18^ ORA refers to participation in formal activities of religious institutions and it is measured on a 6-point Likert type scale by asking the question: “How often do you attend church or other religious meetings?” NORA refers to religious activities performed in private and it is measured by asking the question: “How often do you use your time in religious activities, such as prayer, meditation, reading scriptures, etc.)?” IR is measured by three questions, related to the presence of God as experienced in the lives of people, the relation between religious beliefs and approach to life, and the effort to live religion in all aspects of life. An IR subscale is then obtained by summing the answers to these three items. Possible scale scores on the ORA and NORA dimensions range from 1 to 6, and the IR dimension score ranges from 3 to 15, reflecting lowest to highest religiosity scores, respectively. The Brazilian Portuguese version of DUREL has been validated in different populations.^17,19^

Self-perception of religiosity was measured by the question: “Are you a religious person?,” with the following response options: very religious, moderately religious, a little religious, and not at all religious. Participants were classified into current cigarette smokers, ex-smokers, and those who had never smoked.

### Statistical analysis

Multinomial logistic regression was used to estimate sex and age-adjusted odds ratios (OR) for the relationships between smoking status (current smokers, ex-smokers, or students who had never smoked) and the categories of independent variables (sex, age groups, graduation course, self-perception of health, religion affiliation, and self-perception of religiosity). Sloan et al.^20^ emphasize that variables such as age and sex play a fundamental role in the association between religion and health outcomes and failure to control for these characteristics can lead to biased measures of association. Statistical regression models were therefore used to control for possible confounding effects of sex and age. Adjusted OR were obtained with their respective 95% confidence intervals (95%CI), so that intervals that do not include the value 1 indicate a significant association between the correspondent variable and smoking status (similar to p < 0.05). The VGAM library for R software, version 3.6.2 (R Foundation for Statistical Computing, Vienna, Austria) was used to fit multinomial logistic regression models.

Regression models based on the beta-binomial distribution^21^ were used to assess associations between the three DUREL religiosity dimensions and smoking status, considering age as a covariate. These models are useful for comparing means of discrete variables, assuming non-negative integers from a finite set (such as the DUREL scores). This analysis was stratified by sex because of known differences between women and men in the measures obtained from DUREL. The gamlss library for R software was used for estimation and diagnosis of the beta-binomial regression models.^22^

## Results

The total sample comprised 336 students (83.4% female) who agreed to participate in the research. The non-response rate was 40.5%. Smoking prevalence was 8.3% among females and 12.7% among males.

Table 1 shows OR estimates for the relationships between smoking status and some respondents’ characteristics. We did not find evidence of associations between smoking status and sex, age groups, religion affiliation, or self-perception of religiosity (all of the corresponding 95% confidence intervals include the value 1). Smoking prevalence was highest among Speech Therapy students (15.5%). Ex-smokers have worse self-perceived health. The proportion of smokers among students who do not have a religion, but do believe in God was higher than the proportion of smokers found among those who do have a religion (16.3 and 6.3%, respectively). The highest proportion of smokers was found among agnostics (21.7%).

**Table 1:** Prevalence of current and past smoking by students’ characteristics (n = 336)

	n*	Smoking status (%)			Smokers versus never smoked OR1 (95%CI)^†^	Ex-smokers versus never smoked OR2 (95%CI)^†^
		Current cigarette smokers	Ex-smokers	Never smoked		
Sex						
Women	277	8.3	9.0	82.7	Reference	Reference
Men	55	12.7	9.1	78.2	1.6 (0.64-3.9)	1.1 (0.3-2.9)
Age group (years)						
18-20	163	8.6	11.0	80.4	Reference	Reference
21-25	150	10.0	7.3	82.7	1.1 (0.4-2.3)	0.6 (0.2-1.4)
25 or over	15	13.3	6.7	80.0	1.6 (0.3-7.9)	0.6 (0.1-5.3)
Undergraduate course						
Physiotherapy	138	6.5	7.2	86.2	Reference	Reference
Occupational therapy	40	7.5	12.5	80.0	1.5 (0.3-5.9)	1.8 (0.5-5.8)
Nutrition	74	8.1	9.5	82.4	1.1 (0.3-3.4)	1.4 (0.5-3.9)
Speech therapy	84	15.5	9.5	75.0	3.1 (1.2-7.8)^‡^	1.5 (0.5-4.0)
Self-perception of health						
Good	260	7.7	7.3	85.0	Reference	Reference
Regular	71	14.1	14.1	71.8	2.3 (0.9-5.4)	2.5 (1.1-5.8)^‡^
Has a religion						
Yes	239	6.3	7.5	86.2	Reference	Reference
No, but believes in God	49	16.3	18.4	65.3	3.3 (1.3-8.5)^‡^	3.4 (1.3-8.2)^‡^
Atheist	23	13.0	0	87.0	^§^	^§^
Agnostic	23	21.7	8.7	69.6	3.7 (1.1-11.9)^‡^	1.3 (0.2-6.4)
Religious affiliation						
Catholic	143	4.9	7.7	87.4	Reference	Reference
Evangelical	47	6.4	2.1	91.5	1.3 (0.3-5.0)	0.3 (0.1-2.1)
Spiritist	43	7.0	9.3	83.7	1.5 (0.3-6.1)	1.3 (0.3-4.2)
Are you a religious person?						
Very religious	25	4.0	4.0	92.0	Reference	Reference
Moderately religious	168	6.5	8.9	84.5	1.7 (0.2-14.0)	2.4 (0.3-19.3)
A little religious	72	13.9	8.3	77.8	3.9 (0.4-32.3)	2.4 (0.2-21.3)
Not at all religious	69	11.6	11.6	76.8	2.6 (0.3-23.0)	3.0 (0.4-29.2)

95%CI = 95% confidence interval; OR = odds ratio.

* Some totals do not sum to 336 due to missing observations.

^†^ Sex and age-adjusted odds ratios obtained using multinomial logistic regression models.

^‡^ p < 0.05.

^§^ OR was not calculated due to a zero frequency in the cross-table.

Table 2 shows means and standard deviations (SD) for the dimensions ORA, NORA, and IR, according to smoking status. We found that women who had never smoked tended to have higher mean scores for ORA and IR than women who currently smoke.

**Table 2:** Means and standard deviations for ORA, NORA, and IR subscales of the DUREL, according to students’ smoking status (n = 336)

	Women		Men	
	n	Mean (SD)	n	Mean (SD)
ORA				
Current smokers	22	2.77 (1.27)	7	2.00 (1.29)
Ex-smokers	25	3.00 (1.50)	5	2.60 (1.67)
Never smoked	229	3.41 (1.47)*	43	2.58 (1.42)
NORA				
Current smokers	22	3.05 (1.70)	7	1.57 (1.51)
Ex-smokers	25	3.28 (1.79)	5	3.20 (2.17)
Never smoked	229	3.56 (1.67)	43	2.33 (1.67)
IR				
Current smokers	22	8.23 (3.73)	7	8.43 (3.21)
Ex-smokers	25	10.12 (3.23)	5	8.00 (3.67)
Never smoked	229	10.36 (3.53)*	43	7.63 (3.76)

DUREL = Duke University Religion Index; IR = intrinsic religiosity; NORA = non-organizational religious activity; ORA = organizational religious activity; SD = standard deviation.

* Statistically significant differences from the reference group (current smokers) according to an age-adjusted beta-binomial regression model are marked with an asterisk.

## Discussion

The smoking prevalence rates observed in the present study (8.3% among females and 12.7% among males) are close to the age-standardized prevalence rates of daily smoking among the overall Brazilian population (respectively, 8.2 and 12.6%).^1^ In other studies, the smoking prevalence among Brazilian undergraduate students studying health sciences varies over a very wide range: 3.1% in the Federal District,^6^ 3.2% in Northeast Brazil,^8^ 5.7% in Campina Grande (Northeast region),^5^ 8.9% in Recife (Northeast region),^7^ and 21.1% in Várzea Grande (Mid-West region).^9^ These regional differences suggest that smoking habits may be strongly associated with cultural and social factors.

Table 1 shows a lower smoking prevalence among students who have a religion (6.3%) than among those who do not have a religion, but do believe in God (16.3%), and this percentage is similar among atheists (13.0%). Table 2 shows a significant effect of ORA on smoking status, but there is no evidence of a NORA effect on smoking. These results suggest that it is not belief in God that has an effect on smoking, but the fact of belonging to a religious association. In addition, our results do not show significant differences in smoking prevalence between Catholics, Evangelicals, and Spiritists.

As pointed by Gryczynski & Ward,^23^ the relationship between religiosity and substance use is complex, nuanced, and remains only partially understood. Since the results of the present study suggest that organizational religious activities may play a more important role in the relationship between religiosity and smoking than faith, belief, and other aspects internal to the students, it is recommended that future studies in this field examine the role of mediating and moderating variables in order to shed light on this question. Assuming a religious home environment, the perceived disapproval of smoking from parents and from a social network based on members of a religious institution can strongly mediate the relationship between religiosity and cigarette use. Although studies have suggested that people with higher religious attendance would benefit from social support, well-being, less depressive symptoms, and meaning and would be more committed to healthy behaviors, including less tobacco consumption,^24,25^ our hypothesis does not consider the religion itself as an influence on the individual, rather the social norms stimulated by the different religious organizations.

Although a number of studies have reported significant associations between religious variables and smoking among Brazilian health sciences students,^5-10^ their primary focus was not on religion and religiosity. Sloan & Bagiella^26^ cautioned that conclusions from articles not explicitly about religion and health could be biased, since the correspondent analyses tend to lack controls for confounders and covariates. VanderWeele^27^ also exposed the need for extensive control of confounding factors in studies about the effects of religion on health, in addition to highlighting the importance of longitudinal designs and large sample sizes as fundamental criteria for assessing evidence for causality. In this regard, although the present study is focused on religious involvement, two major limitations should be mentioned. The first limitation is that the cross-sectional design makes it impossible to verify causal relations between variables. The second limitation concerns the fact that the relationship between religiosity and smoking was only controlled for sex and age. Results were not controlled for other potential confounders such as income, social support, depressive symptoms, ethnicity, socioeconomic status, or health status.^20^ However, the data were obtained from a specific sample of students at a public university and we believe that this group is relatively homogeneous with respect to cultural and socioeconomic levels, which to some extent can reduce confounding effects.

This study has other limitations. It was conducted with students from a single university in Southeast Brazil. Additional information such as previous religious affiliations and how long the students have been professing their current religion were not captured. Despite these shortcomings, the results provide evidence of an influence of affiliation on the students’ health practices, showing that a lifestyle motivated by religious concerns tends to strongly influence smoking habits. The importance of these results is related to the fact that a number of students studying healthcare subjects have health habits that are not exclusively motivated by the technical knowledge acquired in their undergraduate courses, since there is possibly a strong influence from social norms stimulated by religious institutions on their health attitudes, knowledge, and practices. This fact should be considered in the design of institutional programs aimed at reducing the number of smokers.

## References

[1] . GBD 2015 Tobacco Collaborators. Smoking prevalence and attributable disease burden in 195 countries and territories, 1990-2015: a systematic analysis from the Global Burden of Disease Study 2015. Lancet. 2017;389:1885-906. 10.1016/S0140-6736(17)30819-X PMC5439023 28390697

[2] . Szklo AS, de Souza MC, Szklo M, de Almeida LM. Smokers in Brazil: who are they? Tob Control. 2016;25:564-70. 10.1136/tobaccocontrol-2015-052324 26292700

[3] . Pinto MT, Pichon-Riviere A, Bardach A. The burden of smoking-related diseases in Brazil: mortality, morbidity and costs. Cad Saude Publica. 2015;31:1283-97. 10.1590/0102-311X00192013 26200375

[4] . Mostardinha AR, Pereira A. Alcohol and tobacco consumption associated factors among college students: a review. Psychol Comm Health. 2019;8:85-98.

[5] . Granville-Garcia AF, Sarmento DJ, Santos JA, Pinto TA, de Sousa RV, Cavalcanti AL. Smoking among undergraduate students in the area of health. Cien Saude Colet. 2012;17:389-96. 10.1590/s1413-81232012000200013 22267034

[6] . Monteiro LZ, Varela AR, Carneiro MLA, Alves LR, Góis RFG, Lima TB. Use of tobacco and alcohol among health care students. Rev Bras Promoc Saude. 2018;31:1-9.

[7] . Leonel AC, Bonan PR, de Castro JF, Dos Anjos Pontual A, Ramos-Perez FM, Feitosa DS, et al. Tobacco use, attitudes, knowledge, and perception about smoking cessation counseling among Brazilian dental students: a cross-sectional study. J Cancer Educ. 2019 Aug 29. doi: 10.1007/s13187-019-01610-6. Online ahead of print. 10.1007/s13187-019-01610-6 31463811

[8] . Granja GL, Lacerda-Santos JT, Brilhante DM, Nóbrega IS, Granville-Garcia AF, Caldas AF Jr, et al. Smoking and alcohol consumption among university students of the healthcare area. J Public Health. 2018;28:45-52.

[9] . Botelho C, da Silva AM, Melo CD. Smoking among undergraduate health sciences students: prevalence and knowledge. J Bras Pneumol. 2011;37:360-6. 10.1590/s1806-37132011000300013 21755192

[10] . Stramari LM, Kurtz M, da Silva LC. Prevalence of and variables related to smoking among medical students at a university in the city of Passo Fundo, Brazil. J Bras Pneumol. 2009;35:442-8. 10.1590/s1806-37132009000500009 19547853

[11] . Ameri Z, Mirzakhani F, Nabipour AR, Khanjani N, Sullman MJ. The relationship between religion and risky behaviors among Iranian university students. J Relig Health. 2017;56:2010-22. 10.1007/s10943-016-0337-1 27990616

[12] . Nabipour AR, Alizadeh A, Saadat-Hosseini M, Mansouri Z, Shamsoddini L, Nakhaee N. Correlates of waterpipe smoking among Iranian university students and the role of religiosity. Int J Psychiatry Med. 2016;51:494-507. 10.1177/0091217417696735 28629297

[13] . Isralowitz R, Reznik A, Sarid O, Dagan A, Grinstein-Cohen O, Wishkerman VY. Religiosity as a substance use protective factor among female college students. J Relig Health. 2018;57:1451-7. 10.1007/s10943-017-0521-y 29110202

[14] . Pule HM, Mashegoane S, Makhubela MS. Intrinsic religiosity and health risk behaviours among black university students in Limpopo, South Africa. J Relig Health. 2019;58:937-48. 10.1007/s10943-017-0555-1 29318436

[15] . Gomes FC, de Andrade AG, Izbicki R, Almeida AM, de Oliveira LG. Religion as a protective factor against drug use among Brazilian university students: a national survey. Braz J Psychiatry. 2013;35:29-37. 10.1016/j.rbp.2012.05.010 23567597

[16] . Zanetti ACG, Cumsille F, Mann R. The association between the use of alcohol, marijuana and cocaine and the sociodemographic characteristics of university students of Ribeirão Preto, Brazil. Texto Contexto - Enferm. 2019;28:e110.

[17] . Taunay TCDE, Gondim FAA, Macêdo DS, Moreira-Almeida A, Gurgel LA, Andrade LMS, et al. Validity of the Brazilian version of the Duke Religious Index (DUREL). Rev Psiquiatr Clin. 2012;39:130-5.

[18] . Koenig HG, Büssing A. The Duke University Religion Index (DUREL): a five-item measure for use in epidemiological studies. Religions. 2010;1:78-85.

[19] . Lucchetti G, Lucchetti AL, Peres MF, Leão FC, Moreira-Almeida A, Koenig HG. Validation of the Duke Religion Index: DUREL (Portuguese version). J Relig Health 2012;51:579-86. 10.1007/s10943-010-9429-5 21107911

[20] . Sloan RP, Bagiella E, Powell T. Religion, spirituality, and medicine. Lancet. 1999;353:664-7. 10.1016/s0140-6736(98)07376-0 10030348

[21] . Najera-Zuloaga J, Lee DJ, Arostegui I. Comparison of beta-binomial regression model approaches to analyze health-related quality of life data. Stat Methods Med Res. 2018;27:2989-3009. 10.1177/0962280217690413 29298606

[22] . Stasinopoulos DM, Rigby RA. Generalized additive models for location scale and shape (GAMLSS) in R. J Stat Softw. 2007;23:1-46.

[23] . Gryczynski J, Ward BW. Social norms and the relationship between cigarette use and religiosity among adolescents in the United States. Health Educ Behav. 2011;38:39-48. 10.1177/1090198110372331 21189421

[24] . Thoresen CE. Spirituality and health: is there a relationship? J Health Psychol. 1999;4:291-300. 10.1177/135910539900400314 22021598

[25] . Oman D, Thoresen CE. Does religion cause health? Differing interpretations and diverse meanings. J Health Psychol. 2002;7:365-80. 10.1177/1359105302007004326 22112748

[26] . Sloan RP, Bagiella E. Claims about religious involvement and health outcomes. Ann Behav Med. 2002;24:14-21. 10.1207/S15324796ABM2401_03 12008790

[27] . VanderWeele TJ. Causal effects of religious service attendance? Soc Psychiatry Psychiatr Epidemiol. 2017;52:1331-6. 10.1007/s00127-017-1434-5 28956085

